# Research impact: neither quick nor easy

**DOI:** 10.1186/s12916-015-0501-6

**Published:** 2015-10-14

**Authors:** Sally Redman, Abby Haynes, Anna Williamson

**Affiliations:** The Sax Institute, Level 13 Building 10, 235 Jones Street, Ultimo, NSW 2007 Australia

**Keywords:** Funding, Health policy, Knowledge translation, Partnerships, Research impact

## Abstract

Greenhalgh and Fahy’s paper about the 2014 Research Excellence Framework provides insights into the challenges of assessing research impact. Future research assessment exercises should consider how best to include measurement of indirect and non-linear impact and whether efforts in knowledge transfer and co-production should be explicitly recognised. Greenhalgh and Fahy’s findings also demonstrate that the structure of the assessment exercise can privilege certain kinds of research and may therefore miss some research that has a high impact on policy and practice. There are a growing number of courses, tools, and funding models to assist researchers in making an impact, although as yet there is little evidence about whether these approaches work in practice.

Please see related article: http://www.biomedcentral.com/1741-7015/13/232.

## Background

The potential of research evidence to improve health outcomes and optimise resource use is widely recognised by governments [1, 2]. There is a burgeoning research literature about the factors that influence research use by policymakers [3–5] and in clinical practice [6]. Arguably, there has been relatively less attention paid to understanding the kinds of research and researchers that have influence.

## Measuring research impact

Greenhalgh and Fahy’s paper [7] will stimulate discussion about the ways in which research has impact and what this means for assessment processes such as the 2014 Research Excellence Framework (REF2014). They point out that the REF2014 encourages reporting of direct and relatively short-term research impacts. While these are important to capture, there is a widely accepted view that indirect impacts driven through non-linear mechanisms should also be considered [8, 9]. Weiss, for example, in her seminal paper [10], describes a common process by which research enters policy as ‘*enlightenment*’, in which ‘*concepts and theoretical perspectives*’ accumulate to influence policy. Indeed, there is growing analysis of approaches to assessing research impact, most of which draw attention to the need for nuanced thinking. For example, commentators have noted the need to pay attention to interactions and feedback loops [11], relative contribution [12, 13], the policy context [14, 15], and the differences between research uptake, use, and impact [13].

As experience with expert panel-based research assessment exercises accrues, it will be valuable to better understand how these panels make decisions, whether the decisions are replicable, and whether researchers and policymakers make similar assessments. In considering methods of assessment, a distinction can also be made between the impact of a piece of research and the impact of a researcher over his or her lifetime. This lifetime impact may prove to be a more dependable assessment of researcher contribution.

Greenhalgh and Fahy’s paper [7] also raises questions about the kinds of research that influence policy and practice. They report that most of the case studies included in the REF2014 used quantitative methods (randomised trials, systematic reviews, longitudinal cohort studies, and modelling studies) and conclude that the format of the REF2014 privileges certain types of research. However, policymakers must consider local applicability, scalability, generalisability, cost, and unintended side effects. Consequently, they will be interested in questions focused towards what works, for whom, in what circumstances, in what respects, how, and why. How can the program be adapted to help it work better? How should elements in the wider system be modified to help [8, 16]? Inevitably, these questions will require mixed methods research and likely the development of new methodologies for working in more applied ways. Impact assessment exercises should be designed to capture these kinds of research.

## The role of researchers in knowledge translation

This analysis of REF2014 also raises interesting questions about co-production of knowledge and how researchers think about their role in relation to knowledge translation. Greenhalgh and Fahy report that, of the 162 case studies, 82 ‘*described strong and ongoing linkages with policymakers, but only 38 described targeted knowledge translation activities. In 40 case studies, no active efforts to achieve impact were described*’. They call for clearer reporting of processes and activities oriented to achieving an impact.

Researchers are ambivalent about their role in knowledge translation. In a study exploring the strategies that thirty-six ‘influential’ researchers used to influence public health policy [17] many described high levels of engagement and co-production, but others felt that ‘*the independence of research is compromised when policymakers are involved in its development*’ and were more comfortable with the archetype of the disinterested scientist who sees ‘*the accumulation of institutionally certified knowledge as an end in itself*’ [18]. This study [17], like many others, notes the considerable cost associated with knowledge translation and co-production (e.g. [19, 20]).

However, policymakers place considerable value on what might be called partnering skills in determining whether to work with researchers, citing, pragmatism, understanding government, authenticity, and collaboration and communication skills as among the criteria they used to assess trustworthy researchers [21]. There is also evidence that researchers are increasingly using knowledge translation strategies and that these appear to pay off. For example, Newson et al. [22] examined a sample of 50 intervention studies funded by the Australian National Health and Medical Research Council and found that dissemination actions by researchers, particularly trying to engage with policymakers or decision makers, and translational inputs such as protocols, treatment manuals, and training materials, were important in influencing whether the research had an impact.

## Supporting researchers in knowledge translation

Despite these complexities, assessment of impact in exercises like the REF2014 is valuable because it demonstrates to governments and the community the value of investing in research; it can also encourage researchers and their institutions to think about the end use of their research and to get better at maximising its impact. More could be done to support researchers in these efforts. For example, in recent years, a number of courses have been established to help researchers build skills in working with policy agencies, although to date there is only limited evidence of their value. A Nigerian course evaluation suggested improvements in participants’ understanding of the policy process and self-reported capacity to adapt research for policy (significance not tested) [23]. A multicomponent evaluation of the Canadian Summer Institute’s course [24] is underway; however, process data and reports from satisfied students suggest a successful model [25, 26]. A brief report on the first two rounds of the Public Health Insight (Australia) group’s one-day Knowledge Translation course suggested the course was considered relevant and useful by participants and that gains were made in understanding and confidence in regards to the skills taught [27]. Policymakers report that the extent to which researchers understand the policy environment is important [21]; therefore, opportunities for researchers to work in the policy setting and vice versa may be particularly valuable (e.g. [28]) and placements are sometimes included in training programs.

Tools and resources might also be valuable in speeding up the rate of learning about how to work effectively with policy agencies. A growing number are available [29–32], although again, there is little evidence about their use or value.

There is also a role for different models of funding research and knowledge translation that offer support for mutual knowledge exchange and co-production, such as the NIHR CLARHRCs in the UK, the Canadian Knowledge to Action funding program, and the NHMRC partnership grants in Australia [33]. Again, there is as yet relatively little investigation of whether these kinds of funding increase impact; an Australian study examined impact of a policy-driven research funding program run by a state health department – while no comparative data were provided, the level of impact was high and arguably higher than one might find in an investigator-initiated scheme [34].

## Conclusions

The formal assessment of research impact is in its infancy. Greenhalgh and Fahy’s paper [7] will contribute to discussions about how to improve assessment exercises in the UK and internationally. We must be sure that in the process of attempting to measure impact we retain a sophisticated and contextualised perspective, and that we support researchers to work effectively with policy and practice agencies.
